# Capsule neural network and its applications in drug discovery

**DOI:** 10.1016/j.isci.2025.112217

**Published:** 2025-03-14

**Authors:** Yiwei Wang, Binyou Wang, Jun Zou, Anguo Wu, Yuan Liu, Ying Wan, Jiesi Luo, Jianming Wu

**Affiliations:** 1 School of Basic Medical Sciences, Southwest Medical University, Luzhou 646000, China; 2 Key Laboratory of Medical Electrophysiology, Ministry of Education & Medical Electrophysiological Key Laboratory of Sichuan Province, Institute of Cardiovascular Research, Southwest Medical University, Luzhou 646000, China; 3 State Key Laboratory of Biotherapy and Cancer Center, West China Hospital, Sichuan University, Chengdu 610041, China; 4 Sichuan Key Medical Laboratory of New Drug Discovery and Druggability Evaluation, Luzhou Key Laboratory of Activity Screening and Druggability Evaluation for Chinese Materia Medica, School of Pharmacy, Southwest Medical University, Luzhou 646000, China

**Keywords:** Health sciences, Medicine, Computer science, Artificial intelligence applications

## Abstract

Deep learning holds great promise in drug discovery, yet its application is hindered by high labeling costs and limited datasets. Developing algorithms that effectively learn from sparsely labeled data is crucial. Capsule networks (CapsNet), introduced in 2017, solve the spatial information loss in traditional neural networks and excel in handling small datasets by capturing spatial hierarchical relationships among features. This capability makes CapsNet particularly promising for drug discovery, where data scarcity is a common challenge. Various modified CapsNet architectures have been successfully applied to drug design and discovery tasks. This review provides a comprehensive analysis of CapsNet’s theoretical foundations, its current applications in drug discovery, and its performance in addressing key challenges in the field. Additionally, the study highlights the limitations of CapsNet and outlines potential future research directions to further enhance its utility in drug discovery, offering valuable insights for researchers in both computational and pharmaceutical sciences.

## Graphical abstract

**Figure:**
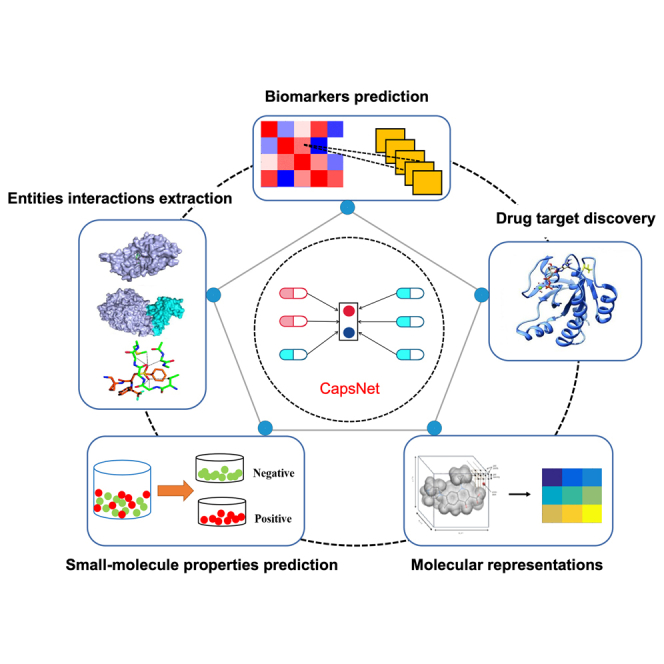

## Introduction

Conventional drug discovery and innovation processes are expensive, time-consuming, and are characterized by a significant risk of failure.^1^ For decades, computer-aided drug design (CADD) has been instrumental in accelerating the drug discovery process. With more complexity of disease mechanisms and the increase of the chemical universe, traditional CADD is unable to handle such complicated problems satisfactorily. As a subset of artificial intelligence (AI), deep learning has demonstrated its capability to expedite the drug discovery process through its ability to unearth new and critical insights from extensive and intricate datasets.^2^ AI-driven novel CADD strategies, in contrast to conventional methods, place a greater emphasis on data rather than the targets.^3^ Deep learning algorithms have been applied to drug discovery tasks, yielding impressive results. The effectiveness of cutting-edge deep learning models heavily relies on the presence of large, accurately labeled training datasets. However, gathering, annotating, and verifying substantial volumes of data in the realm of deep learning-supported drug discovery and design represents a costly and labor-intensive endeavor. The scarcity of large data with high quality is one of the obstacles to the application of deep learning methods in drug discovery. To address the issue of limited data, a variety of data augmentation techniques, such as random rotations, translations, and flips, are frequently utilized. However, these techniques are not appropriate for small molecules and proteins with various physicochemical and biological information in the drug discovery pipeline. Several innovative approaches, including transfer learning, multitask learning, one-shot and few-shot learning, zero-shot learning, and their combinations, are anticipated to address this challenge effectively. It is also worth noting that these learning methods come with their own set of limitations. For example, catastrophic forgetting is a major limitation inherent in transfer learning and one/few-shot learning.^4^ In 2017, Hinton et al. introduced a groundbreaking deep learning structure, CapsNet, targeting the issue of data loss attributed to the pooling approach employed by convolutional neural networks (CNNs).^5^ CapsNet has been applied in many practical problems and has proven utility in improving performance for complex, small datasets. Inspired by the strengths of CapsNet in small dataset scenarios, its application has extended to drug discovery and related areas where it has achieved performance that is either superior to or on par with sophisticated traditional methods.

At present, the applications of CapsNet in drug discovery are mostly focused on developing high-performance prediction models based on various benchmark tasks. The applications of CapsNet on innovative drugs remain sparse. Thus, a deeper understanding of CapsNet models applied in drug-related studies can enhance the interpretation of these models and facilitate the development of advanced strategies for innovative drug discovery.

The primary objective of this review is to underscore the current applications of CapsNet in drug discovery while also examining the prospects and challenges associated with its implementation. This work aims to serve as a valuable resource for researchers in the field. To the best of our knowledge, this paper is the first comprehensive review of CapsNet and its applications in drug discovery and design.

## Overview of CapsNet

### Concept

**Figure 1:**
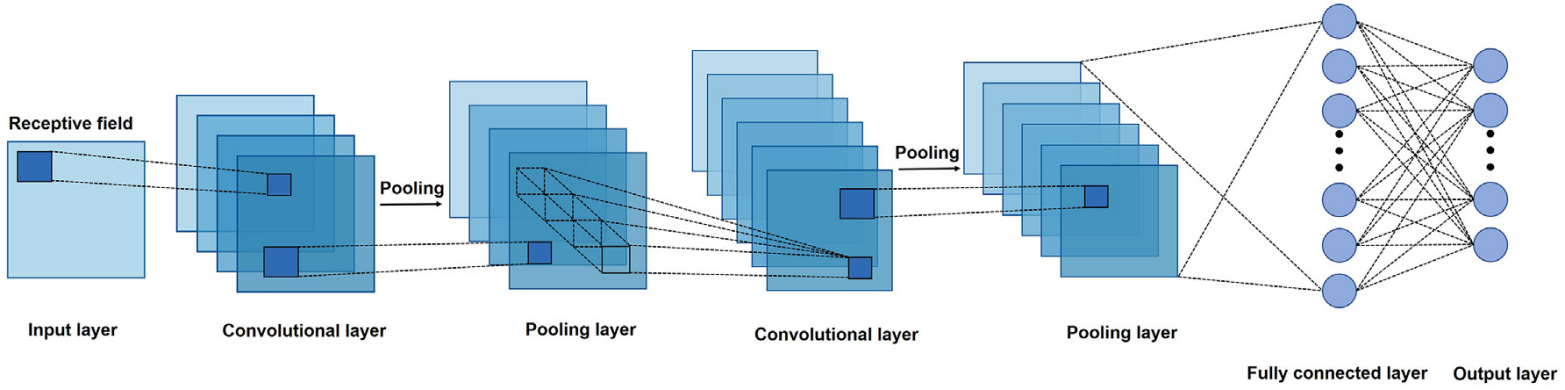
Basic architecture of a CNN The detailed descriptions for the theoretical of CNN were presented in ref.^5^

**Figure 2:**
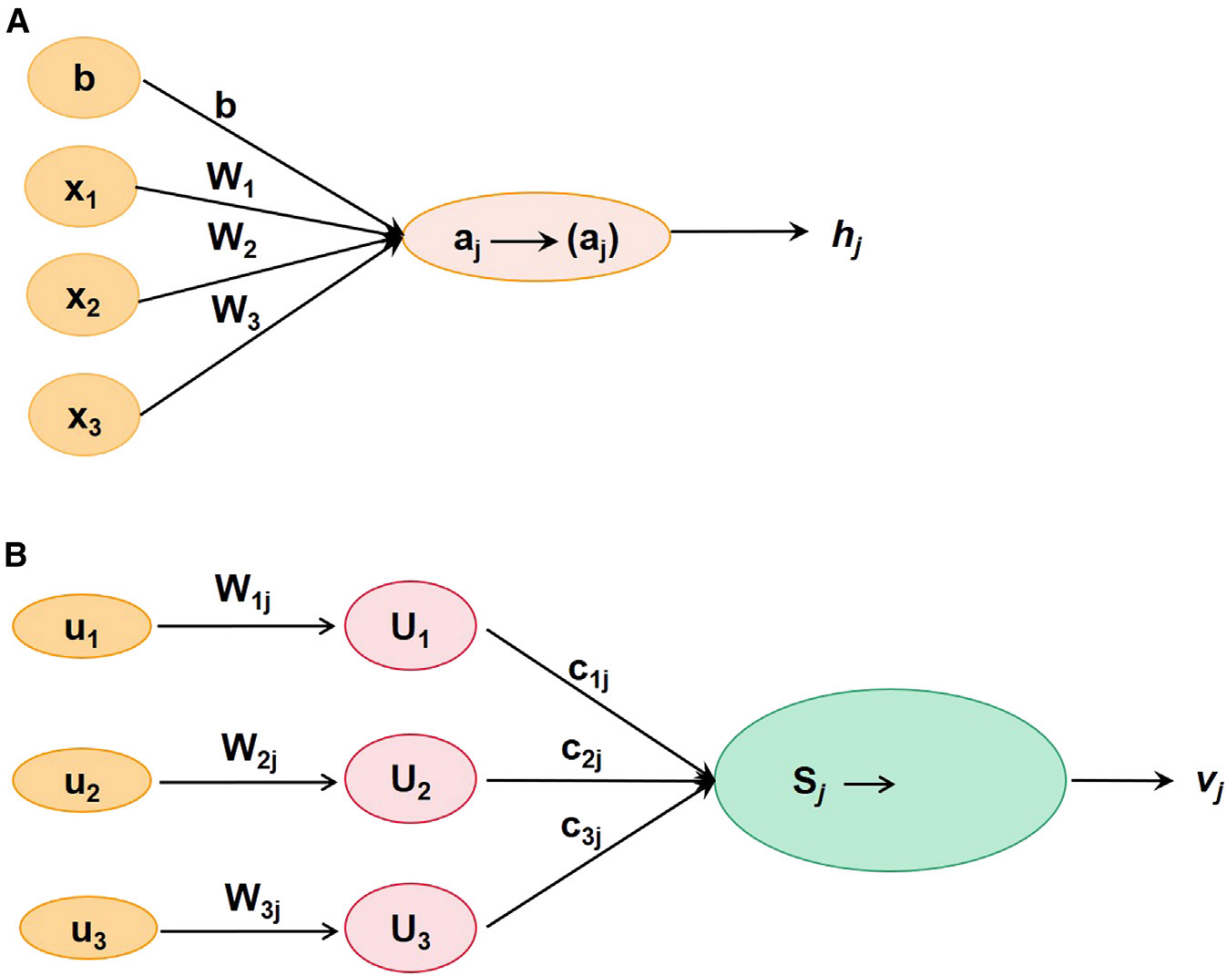
Comparison between scalar neurons and vector neurons (A) Scalar neurons in conventional deep learning. (B) Vector neurons in CapsNet.

In recent years, CNN has become one of the most utilized types of deep learning networks.^6^ The architecture of CNN, illustrated in Figure 1, comprises layers of convolution, pooling, and fully connected components. While CNN has achieved success in various applications, it has significant limitations. The pooling operation often fails to maintain precise spatial information and translation equivariance. Moreover, CNN struggles to capture hierarchical relationships between basic and complex features, which typically requires large amounts of training data for optimal performance. These challenges underscore the need for improvements in CNN architecture. To address these limitations, Hinton et al. introduced an innovative neural network known as the “capsule network”. This term was derived from the notion of a “capsule” initially presented in 2011.^7^ The traditional deep neural network is made up of scalar neurons (Figure 2A), whereas CapsNet is constructed with capsules. Each capsule consists of a set of vector neurons (Figure 2B). The length of a vector signifies the likelihood of an entity’s presence, while the direction of the vector denotes the entity’s state, namely “instantiation parameters”. The parameters of instantiation may vary across different viewpoints, a characteristic known as the equivariant property of CapsNet. To realize the equivariant property, the capsule would produce a vector with small change as the output as the object changes slightly. The equivariant property enables CapsNet to learn the same object from various locations and viewpoints. As a result, CapsNet can not only identify new objects but also understand their relationships. This advantage enables CapsNet to learn effectively from limited training data, requiring fewer samples to form robust representations compared to other deep learning architectures.

### Basic principles

There are three existing general methods of capsule implementation reported in the literature: transforming auto-encoders,^7^ vector capsules based on dynamic routing,^5^ and matrix capsules based on expectation maximization routing.^8^ Among these approaches, the dynamic routing-based methodology is the most utilized in a variety of studies, particularly in drug discovery. Consequently, this method was selected for in-depth discussion in our review, where it is referred to as the original CapsNet.

Both the input and output of CapsNet are vectors, as illustrated in Figure 2B. They are generated as follows: (1) The original input vector *u* is translated into a new input vector *U*_*j|i*_ by a weight matrix; (2) *U*_*j|i*_ is multiplied by the coupling coefficient *c*_*ij*_; (3) the total input vector for capsule *j* (*s*_*j*_) is achieved by summing all the weighted input vectors; and (4) *s*_*j*_ is transformed into the output vector *v*_*j*_ by a nonlinear vector function.

**Figure 3:**
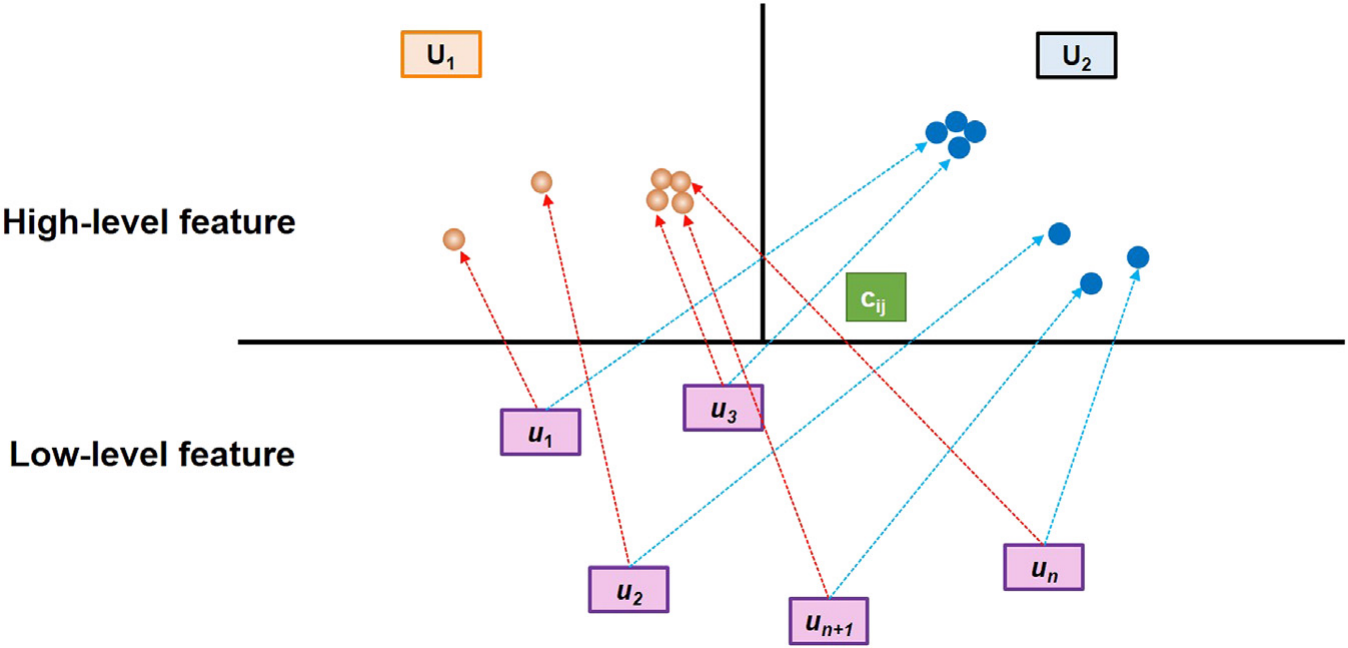
Dynamic routing and hierarchical feature integration mechanism in CapsNet Transmission procedure between a high-level capsule and low-level capsule: (1) Unlike the fixed connections in CNN, low-level capsules transmit information to high-level capsules using a dynamic routing mechanism. Each low-level capsule determines its connection to high-level capsules based on its activation level and relevance. (2) High-level capsules receive information from multiple low-level capsules and integrate this data to form more complex feature representations. These complex features encompass not only the presence of low-level features but also their combinations and spatial relationships.

We present a specific example to describe the basic principles of CapsNet in detail. There are *N* lower-level features, namely $u_{1}$, $u_{2}$, …, $u_{N}$, and the higher-level features are defined as *U*. The workflow consists of four steps. In step 1, matrix transformation is implemented. The low-level features can be transformed into high-level features via the relationships between the low-level and high-level features:

(Equation 1) $$u_{j|i}=w_{ij}\cdot u_{i}$$

where $u_{i}$ represents the low-level feature; $u_{j|i}$ indicates the high-level feature inferred from the low-level feature; and $w_{ij}$ is the spatial relationship between low-level and high-level features. A specific high-level feature will manifest in a certain position if the predicted outcomes of the low-level features are in strong agreement with one another. In the second step, the weights serve as the input. Figure 3 depicts a low-level feature determining to forward its output to a high-level feature, a decision achieved through the modification of coupling coefficients.

(Equation 2) $$c_{ij}\times u_{j|i}$$

where $c_{ij}$ are the coupling coefficients that are decided by the dynamic routing process and computed by the *softmax* function as follows:

(Equation 3) $$c_{ij}=\frac{\exp\left(b_{ij}\right)}{\sum_{k}\exp\left(b_{ik}\right)}$$

The initial logits *b*_*ij*_ are the prior probabilities that the lower-level capsule *i* should be coupled to the higher-level capsule *j*. In step 3, the weighted sum is computed. A weighted summation of all high-level features derived from step 2 is carried out:

(Equation 4) $$s_{j}=\sum_{i}c_{ij}u_{j|i}$$

where *s*_*j*_ represents the agreement output of the low-level features, and the $c_{ij}$ between low-level capsule *i* and high-level capsule *j* is 1, meaning all the outputs of capsule *i* are sent to capsule *j*. In step 4, non-linearity is activated. The squash function is applied to activate the agreement output and generate the high-level capsule:

(Equation 5) $$v_{i}=\frac{{\left\|s_{j}\right\|}^{2}}{1+{\left\|s_{j}\right\|}^{2}}\cdot \frac{s_{j}}{\left\|s_{j}\right\|}$$

where *v*_*j*_ is the output vector and in the range of 0 and 1.

### Original architecture

**Figure 4:**
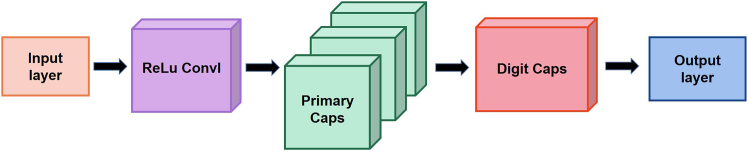
Original architecture of CapsNet (A) Basic architecture; (B)Workflow; (C) Dynamic routing: The process of dynamic routing can be summarized as follows: First, the outputs of the hidden features are encapsulated into capsules, producing high-level features ($u_{j|i}$) and defining the connection probabilities ($b_{j|i}$) between capsules in the previous layer and those in the next layer. Next, these probabilities $b_{j|i}$ are activated using an activation function to generate the coupling coefficients ($c_{j|i}$). Following this, a weighted sum and nonlinear activation are applied to the capsules in the next layer. Finally, $b_{j|i}$ is updated, and this entire process is iterated for a predetermined number of times.

The architecture of the original CapsNet is made up of the convolutional layer, primary capsule layer, and DigitCaps capsule layer (Figure 4A). Unlike conventional layer-to-layer connections, dynamic routing facilitates the transfer of feature information between the PrimaryCaps and DigitCaps layers within CapsNet. This mechanism establishes the relationship between low-level and high-level features, enabling an effective feature transfer process. Specifically, a lower-level capsule transmits its output to a higher-level capsule when they are most relevant to each other. The coupling coefficient represents the agreement among capsules, while the routing-by-agreement algorithm enhances the accuracy of associations between higher-level features and predicted outcomes. Dynamic routing is a unique mechanism within CapsNet that allows for a rapid focus on critical features relevant to predictions, while filtering out elements that have little or no relevance to the outcomes (see Figure 4C). Table 1 provides an overview of the dynamic routing-by-agreement algorithm.

**Table 1:** Algorithm of dynamic routing

Dynamic routing
1: procedure ROUTING $\left({\hat{u}}_{j|i},r,l\right)$
2: for all capsule *i* in layer *l* and capsule *j* in layer (*l+1*): $b_{ij}\leftarrow 0$
3: for *r* iterations do
4: for all capsules *i* in layer *l*: $c_{i}\leftarrow softmax\left(b_{i}\right)$
5: for all capsules *j* in layer (*l+1*): $s_{j}\leftarrow \sum_{i}c_{ij}{\hat{u}}_{j|i}$
6: for all capsules *j* in layer (*l+1*): $v_{j}\leftarrow squash\left(s_{j}\right)$
7: for all capsules *i* in layer *l* and capsule *j* in layer (*l+1*)
8: $b_{ij}\leftarrow b_{ij}+{\hat{u}}_{j|i}\cdot v_{j}$
9: return *v*_*j*_

### Margin loss

Unlike other deep learning networks, the CapsNet classification model can generate multiple vector capsules simultaneously, with each capsule representing a distinct category. The length of each capsule vector indicates the probability of its corresponding category’s presence, such that when a category is active, its capsule length exceeds that of the others. Since multiple entities can be represented concurrently in CapsNet, traditional cross-entropy loss is no longer suitable as the loss function. Instead, margin loss is chosen as the loss function for each class of CapsNet:

(Equation 6) $$L_{k}=T_{k}\;\max\;{\left(0,m^{+}-\left\|v_{k}\right\|\right)}^{2}+\lambda \left(1-T_{k}\right)\max\;{\left(0,\left\|v_{k}\right\|-m^{-}\right)}^{2}$$

where *T*_*k*_ is 1 whenever class *k* is present and otherwise 0. The hyper-parameters m^+^, m^−^, and λ need to be specified prior to the learning process. In the original CapsNet, these parameters were set to m^+^ = 0.9, m^−^ = 0.1, and λ = 0.5, which have been demonstrated to promote training stability and enhance convergence. The value of $\left\|v_{k}\right\|$ represents the output of class *k*. If class *k* exists, $\left\|v_{k}\right\|$ is equal or greater than 0.9; otherwise, the value of $\left\|v_{k}\right\|$ is less than or equal to 0.1.

### Architecture improvements

Although CapsNet has already been successfully deployed in different fields, its original architecture is not suitable for all tasks. As a result, various modified CapsNet architectures have been suggested to enhance performance across different practical tasks. The modifications of the original CapsNet can be categorized into three groups: architecture-based, feature-based, and algorithm-based. These are summarized in Table 2. Based on this innovative architecture, there is increasing interest in combining CapsNet with traditional networks as a robust approach. Deng et al. unveiled an attention-based CapsNet for aspect-level sentiment classification (ABASCap), designed to capture more nuanced text semantics for enhancing classification outcomes.^9^ Jaiswal et al. introduced the Generative Adversarial Capsule Network (CapsuleGAN), where CapsNet takes the place of the standard CNN as the discriminator in the Generative Adversarial Network (GAN) framework.^10^ To enhance the performance of CapsNet on various tasks, adding advanced feature extractors is also an effective way. Yang et al. developed RS-CapsNet, employing Res2Net to harvest features across multiple scales.^11^ Local CapsNet is an enhanced capsule network architecture focused on feature extraction. It employs local capsules to capture features from specific regions of an image. These local features are then integrated into global capsules through dynamic routing, resulting in a high-level representation that effectively captures both the details and local structures of the entire image.^12^ In addition, several novel dynamic routing procedures and loss functions have been proposed to improve robustness and expand the applicability of CapsNet. In 2018, Hinton et al. introduced Expectation Maximization routing (EM routing), with the objective of iteratively refining the connection weights between capsules to enhance the understanding of their interrelationships.^8^ Wang et al. proposed a simple and stable dynamic routing approach by using entropy regularization.^13^ Ren et al. introduced a novel routing algorithm utilizing k-means clustering to determine the allocation of credit between capsules at lower and upper levels.^14^ The existing loss functions of CapsNet cannot be directly reused sometimes, and accordingly, the loss function is another direction for improvement. Srivastava et al. introduced a single model capsule network with focal loss to alleviate the imbalance problem of the classification task.^15^ To better learn multiple relations, Zhang et al. proposed a sliding-margin loss function, and embedded it into CapsNet.^16^ A novel CapsNet with survival loss was proposed by Tang et al. to perform survival analysis.^17^

**Table 2:** Three categories of modified CapsNet models

Method	Description	Examples	Example of application in drug discovery
Architecture-based	Combining CapsNet with other advanced classical networks to improve performance.	ABASCap,^9^ CapsuleGAN,^10^ AA-GCN.^18^	CapsNet integrated with various attention mechanisms for CPI extraction,^19^ DDI relation classification,^20^ prediction of the sensitivity of cancer cell lines,^21^ respectively.
Feature-based	Extracting useful features from different aspects to enhance the representation ability of the model.	RS-CapsNet,^11^ Cabor capsule network,^22^ CapsGNN,^23^ Caps-HAGKT.^24^	Protein and molecule datasets have different properties that are being learned and tested.^25^^,^^26^^,^^27^
Algorithm-based	Using novel dynamic routing or a loss function to replace the original ones to suit various tasks.	EM-Routing,^8^ DeepCaps,^28^ DR-CapsGNN,^29^ CapsGNNEM.^30^	\

AA-GCN, adaptive attention graph capsule network; CapsGNN, capsule graph neural network; Caps-HAGKT, knowledge-tracking method based on a multi-hierarchical capsule graph neural network; DeepCaps, deep capsule network architecture; DR-CapsGNN, a dual-routing capsule graph neural network; CapsGNNEM, capsule graph neural network with EM routing.

It is expected that more advanced and improved schemes will be proposed for designing new CapsNet models with superior performance and more interpretable results; hence, more complex, and specific questions will be addressed by CapsNet soon.

### Comparison with CNN in drug discovery

Given that CapsNet represents an advancement over traditional CNN models, it is crucial to conduct a comparison with the fundamental CNN architecture. Different from the traditional CNN-based methods, CapsNet is made up primarily capsules. A capsule comprises neuron vectors, serving as a distinctive and potent building block to more effectively model the varied relationships within the neural network’s internal representations. For instance, in a study focused on predicting posttranslational modification (PTM) sites, the dimension of a capsule was indicative of the likelihood of PTM occurrence, while the capsule’s orientation revealed the unique sequence characteristics of the substrates within various PTM subclasses.^31^ Unlike CNN, CapsNet can not only describe the probability of the presence of the PTM of interest but also encode information about its properties such as pose, deformation, and other characteristics. In a different study focusing on predicting small-molecule properties, the length of the capsule indicated the likelihood of the presence of substructures or properties in a molecule. The orientation could have indicated the relative arrangement of the combination of substructures in the molecule.^32^^,^^33^ Therefore, capsules can provide more relevant information to reinforce the notion of using individual components to understand the entirety.

While CNN relies on the pooling operation to achieve translation invariance, CapsNet offers translation equivariance without the need for such pooling operations. The equivariance enables CapsNet to describe logical relationships between vector components accurately. Using the application of PTM as an illustration, a prediction capsule designated for either a positive or negative outcome gets activated if its forecast aligns with the specific amino acid interactions, facilitated by the dynamic routing mechanism. For molecular activities or properties prediction applications, the equivariance would also work. One-dimensional vector is often used to describe small molecular structures and properties in the drug design task. However, representing logical relationships between vector components is challenging with a one-dimensional vector, and minor changes of a vector component are often overlooked in CNN. The equivariant property of a capsule ensures a precise description of the logical relationships in molecular fingerprints and descriptors, and CapsNet can efficiently learn the characteristics of small molecular structures even if minor changes occur in the vector components.

### Comparison with some advanced deep learning algorithms in drug discovery

In addition to CNN, several advanced deep learning algorithms, such as Recurrent Neural Network (RNN), Graph Neural Network (GNN) and Transformer, have been extensively applied in drug discovery research, showing significant potential and promising results.^34^^,^^35^^,^^36^^,^^37^^,^^38^^,^^39^ To better illustrate the unique value of CapsNet in drug discovery, we continued to directly compare it with RNN, GNN and Transformer. We found that the main differences between the four algorithms primarily lie in their data processing methods. Due to its superior ability to comprehend hierarchical structures and entity relationships within images, CapsNet can learn complex models with less data compared to common deep learning algorithms. However, it remains unclear how this learning approach can be extended to large datasets or more complex models, which might limit the use of CapsNet in data-intensive drug discovery tasks. In contrast, RNN excels at processing sequential data, such as protein sequences or DNA sequences.^40^^,^^41^^,^^42^^,^^43^^,^^44^^,^^45^ However, the challenge of handling long-term dependencies limits the deeper application of RNN in drug discovery. Graph Convolutional Network (GCN) is capable of directly handling graph-structured data, which makes it especially well-suited for molecular representation in drug discovery. It has shown remarkable performance in applications such as drug property prediction and virtual screening.^46^^,^^47^ Nevertheless, GCN still finds it challenging to effectively capture local spatial relationships in complex molecular structures. The Transformer model, with its self-attention mechanism, effectively tackles the challenge of long-range dependencies. Its variants and extensions, such as Bidirectional Encoder Representations from Transformers (BERT) and Generative Pre-trained Transformer (GPT), have shown considerable promise in managing complex bioinformatics data.^48^^,^^49^^,^^50^^,^^51^^,^^52^

In summary, CapsNet excels in processing structured data and image data, whereas RNN is particularly effective for sequential data, and Transformer is well-suited for large-scale text analysis. Each deep learning algorithm has its own specific applications. Therefore, the selection of an appropriate algorithm should be guided by practical considerations, including the specific requirements of the task, the nature of the raw data, and the objectives of the drug discovery process.

## Application of CapsNet for drug discovery

CapsNet was first proposed in 2011, but it did not attract much attention until the comprehensive concepts of CapsNet were proposed in 2017.^18^ The outstanding performance of CapsNet in image recognition has sparked interest among pharmaceutical chemists, leading to its application in various areas of drug discovery and related research. Specifically, CapsNet has been utilized for predicting biomarkers, discovering and identifying targets, creating molecular representations, predicting the activities or properties of small molecules, and extracting interactions between entities. Table 3 summarizes the application of CapsNet for different tasks in drug discovery. We can see that open-source tools play a significant role, allowing researchers to directly learn about the various applications of CapsNet in drug discovery and gain a more intuitive understanding of its capabilities.

**Table 3:** Applications of CapsNet in various drug discovery tasks

Model	Task	URL	Availability
CapsNet-SSP^26^	Predicting for protein biomarkers in saliva	http://www.csbg-jlu.info/CapsNet-SSP/	Free
Capsule network and the encoder–decoder model of the attention mechanism^21^	Predicting the sensitivity of cancer cell lines to both single and combination drug treatments	/	Undisclosed
Capsule networks^53^	Predicting for RAS protein family structures	/	Undisclosed
Capsule networks^54^	Predicting for gamma-turn	http://dslsrv8.cs.missouri.edu/∼cf797/MUFoldGammaTurn/download.html.	Free
DeepCap-Kcr^55^	Predicting for lysine crotonylation (Kcr) site	https://github.com/Jhabindra-bioinfo/DeepCap-Kcr	Free
TF3P^56^	Predicting for three-dimensional small-molecule fingerprints	https://github.com/canisw/tf3p	Free
RBM-CapsNet and Conv-CapsNet^32^	Predicting for hERG blockers/nonblockers	https://github.com/yangsygroup/hERG_CapsNet	Free
Capsule networks^57^	Predicting for disease-related compound	/	Undisclosed
BERT-AttCapsule^19^	Extracting for chemical-protein interaction	/	Undisclosed
Capsule-LPI^25^	Predicting for LncRNA–protein interaction	http://csbg-jlu.site/lpc/predict	Free
AC-Caps^58^	Predicting for RNA-binding protein binding sites of long noncoding	https://github.com/JinmiaoS/AC-Caps	Free
circRB^59^	Predicting for identifying RNA-binding proteins binding sites on circular RNAs	https://github.com/wzf171/circRB	Free
Capsule networks^31^	Predicting protein post-translational modification sites	https://github.com/duolinwang/CapsNet_PTM	Free

CapsNet-SSP, multilane capsule network for predicting human saliva-secretory proteins; DeepCap-Kcr, capsule network for lysine crotonylation (Kcr) site prediction; TF3P, three-dimensional small-molecule fingerprints based on the deep capsular neural network; RBM-CapsNet, Boltzmann machine-capsule network; Conv-CapsNet, convolution-capsule network; BERT-AttCapsule, BERT-based attention-guided capsule networks; BERT, bidirectional encoder representations from transformers; Capsule-LPI, capsule network for identifying LncRNA-protein interaction; LncRNA, long noncoding RNA; AC-Caps, attention based capsule network for predicting RBP binding sites of LncRNA; RBP, RNA-binding protein; circRB, Circular RNA.

### Biomarkers prediction

Accurately measuring and validating biomarkers as key indicators to distinguish between healthy and diseased samples plays a crucial role in disease detection, prognosis formulation, and understanding disease mechanisms.^60^ Machine learning-based approaches for identifying biomarkers and forecasting drug responsiveness have demonstrated efficacy in enhancing clinical outcomes, deepening the understanding of drug mechanisms, and selecting the most appropriate drug for each patient.^61^^,^^62^^,^^63^^,^^64^^,^^65^^,^^66^^,^^67^^,^^68^^,^^69^ Numerous successful predictive models leveraging machine learning, along with their associated biomarkers, have played a crucial role in drug discovery and development.^66^^,^^70^^,^^71^ As an advanced deep learning method, CapsNet offers considerable promise for enhancing biomarker discovery and predicting drug sensitivity. Recently, Du et al. developed an innovative end-to-end model utilizing CapsNet architecture to directly identify saliva-secretory proteins from sequence data. This model effectively demonstrates the ability to accurately discern these proteins based solely on protein sequence information.^26^ Chen et al. merged CapsNet with the attention mechanism’s encoder-decoder model to forecast the sensitivity of cancer cell lines to both single and combination drug treatments, aiming to uncover the direct link between drugs and disease-specific gene expression.^21^ CapsNet can extract more valuable information from cancer cell line characteristics compared to a basic CNN network. The applications discussed above indicate that CapsNet, even with limited input information, has outperformed certain classical machine learning methods in predicting biomarkers.

### Drug target discovery and identification

Drug discovery usually starts by identifying a new target that, when appropriately activated or inhibited, can provide therapeutic benefits with a sufficient safety margin.^72^ Hence, the ultimate success of an innovation drug discovery primarily depends on identifying promising drug targets early in the research phase. However, traditional drug target discovery and identification approaches based on biological experiments are usually difficult and often unfruitful. Recently, a range of machine learning algorithms has been applied across various stages of target discovery and disease subtyping. These include identifying disease-driver genes and microRNAs, predicting alternative splicing, prioritizing novel drug targets, modeling three-dimensional target structures, and assessing druggability.^73^^,^^74^^,^^75^^,^^76^^,^^77^^,^^78^

Understanding the three-dimensional structure of targets is crucial for designing drugs to either inhibit or activate their functions. While numerous deep learning techniques have been used in structural biology,^79^^,^^80^^,^^81^^,^^82^^,^^83^ most of them lack interpretability. For example, CNN fails to consider important spatial hierarchies between lower and higher features, which is crucial in protein structure classification. CapsNet pays particular attention to hierarchical relationships and therefore has great potential to address the above issue in protein structure classification. The initial study focusing on protein classification using CapsNet was conducted by Jesus et al.^84^ This model demonstrated effective categorization of Rat Sarcoma (RAS)family protein structures, achieving higher predictive accuracy compared to baseline CNN. Protein gamma-turn prediction plays an important role in protein three-dimensional structure prediction and protein function studies.^53^ Fang et al. published the first study using deep learning techniques to develop a novel model for gamma-turn prediction.^85^ This model was developed by CapsNet and significantly outperformed the previous best method on the gamma-turn benchmark.

PTM is a crucial chemical process that significantly influences human disease progression.^54^ Recognizing and comprehending PTM are crucial in biological and disease research. Wang et al. proposed a CapsNet for PTM site prediction, which outperformed the baseline CNN architecture and three representative PTM site prediction tools (MusiteDeep, Musite, and ModPred).^31^ Khanal et al. introduced a DeepCap-Kcr model utilizing the CapsNet approach for predicting lysine crotonylation (Kcr) sites.^55^ Their experiments indicated that the proposed model outperformed previous CNN-based models as well as other established architectures, effectively capturing the internal data distribution pertinent to motif detection.

### Molecular representations

**Figure 5:**
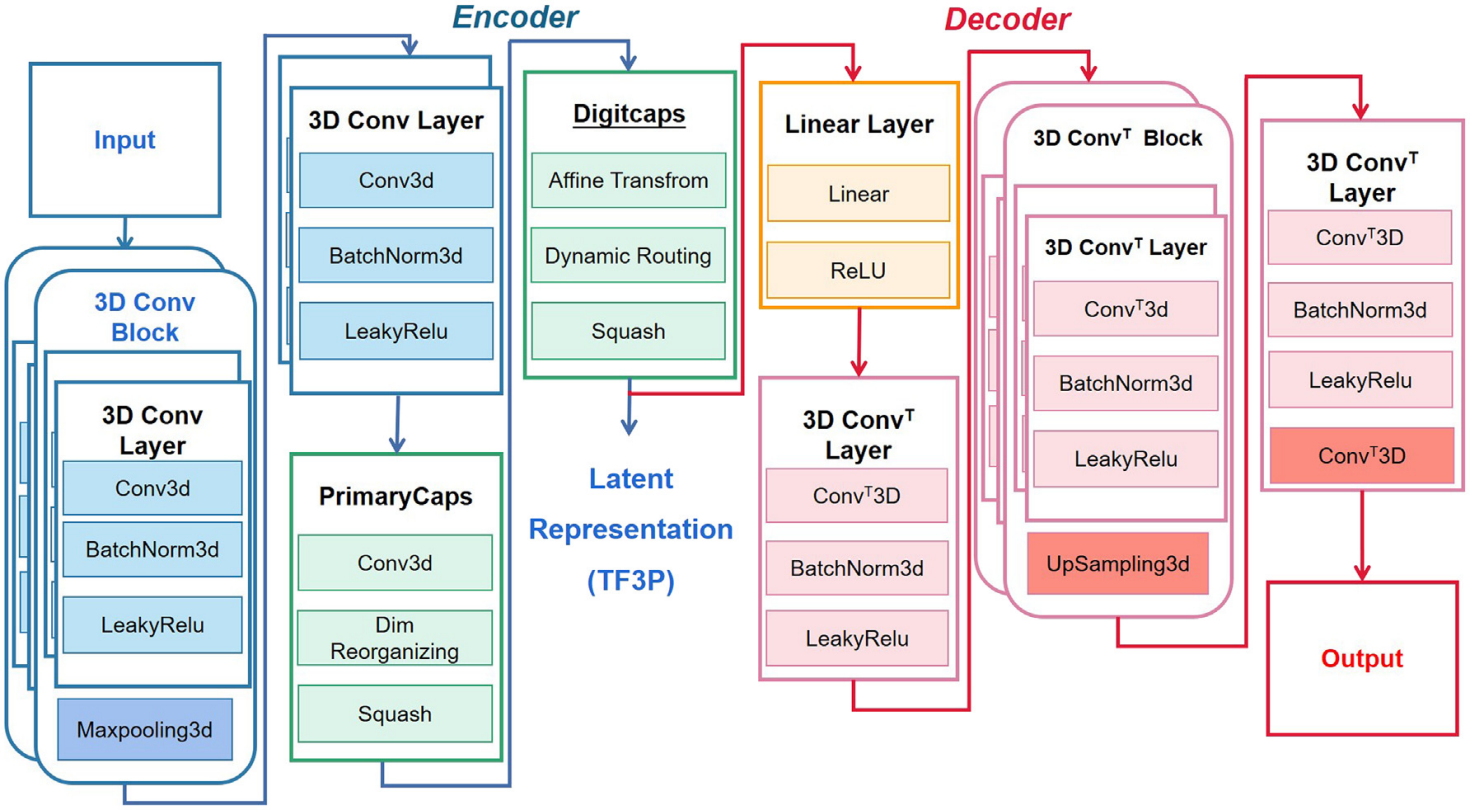
Workflow of three-dimensional small-molecule fingerprints learned by CapsNet Reproduced with permission from.^56^ Copyright 2020 American Chemical Society.

Molecular descriptors are used to characterize properties of a corresponding molecule, and molecular fingerprints are used to characterize structures of molecules that are encoded as binary bit strings. Both molecular descriptors and fingerprints have been pivotal in machine learning-based drug discovery processes. As deep learning advances, more molecular representations are being automatically generated by deep learning models from basic raw inputs.^86^^,^^87^ It’s worth noting that deep learning-generated molecular representations have predominantly concentrated on two-dimensional forms.^87^^,^^88^^,^^89^^,^^90^ However, most of the two-dimensional representations lack unbiased information for an intact molecule, and it is difficult to make the similarity calculation of such fingerprints compatible with statistical models based on similarity. Wang et al. developed a deep learning model utilizing three-dimensional small-molecule fingerprints, leveraging the deep CapsNet architecture (see Figure 5).^56^ As CapsNet can be trained to output the pose matrix of three-dimensional objects, this proposed model demonstrated strong ability to capture three-dimensional structural changes. CapsNet might be more suitable for developing three-dimensional representations than other deep learning methods. Further investigation demonstrated that this new fingerprint of a three-dimensional molecule could be not only be trained without targeting specific predictive tasks but also was compatible with statistical models when applied to ligand similarity modeling.

### Prediction of small-molecule properties

Prediction of small-molecule properties is a prevalent task in drug discovery.^91^ Despite deep learning showing potential in predicting molecular behavior and characteristics,^92^^,^^93^^,^^94^^,^^95^ its superiority in those cases was primarily driven by massive and high-quality datasets.^96^^,^^97^^,^^98^ CapsNet shows promise in predicting molecular properties, especially in the realm of deep learning with limited datasets.

**Figure 6:**
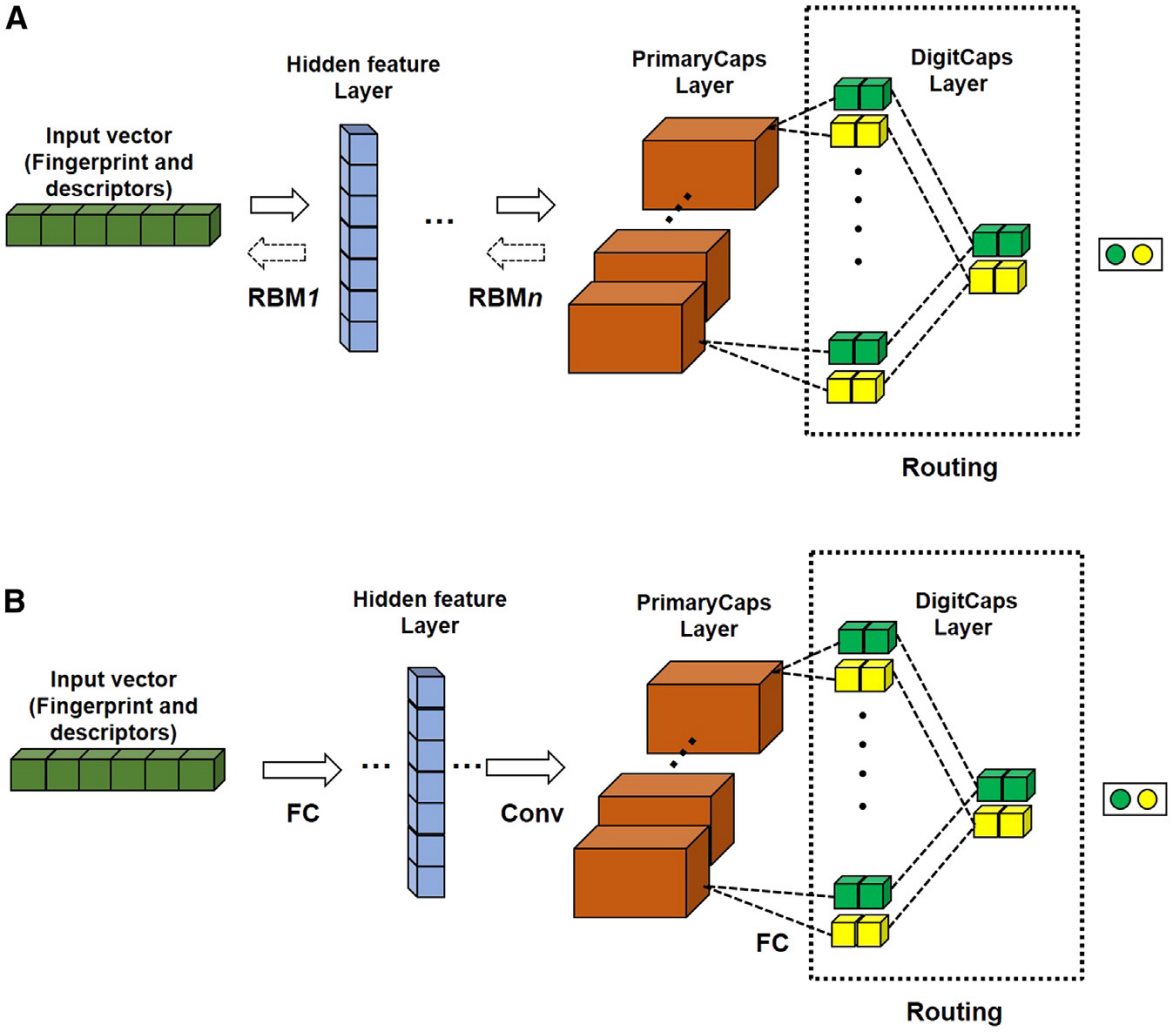
Two novel CapsNet architectures for drug molecular design (A) RBM-CapsNet (B) Conv-CapsNet.

In a pioneering effort, our team developed two novel CapsNet architectures specifically designed for drug design. These architectures were then utilized to predict drug cardiotoxicity and drug-induced carcinogenicity.^32^ We proposed to use restricted Boltzmann machine (RBM) and one-dimensional convolution as the front-end for the new models (RBM-CapsNet and Conv-CapsNet) (Figure 6), which can directly extract the abstract features from the initial feature vectors without changing their dimensions and then preserve maximum information of the input vector features. Compared with other models, both RBM-CapsNet and Conv-CapsNet exhibited the strongest robustness on small datasets for the applications. Machine learning methods for properties prediction frequently faces a significant challenge, namely the training data is usually biased. From the previous results, CapsNet offered a distinct chance to tackle imbalanced data issues.^26^^,^^99^^,^^100^ We established a new multitask framework based on a capsule neural network (multitask CapsNet) to simultaneously assess 12 distinct toxic effects. Our findings indicate that this model could effectively addresses bias issues and presents a novel, accurate, and efficient method for predicting compound toxicities.^101^

**Figure 7:**
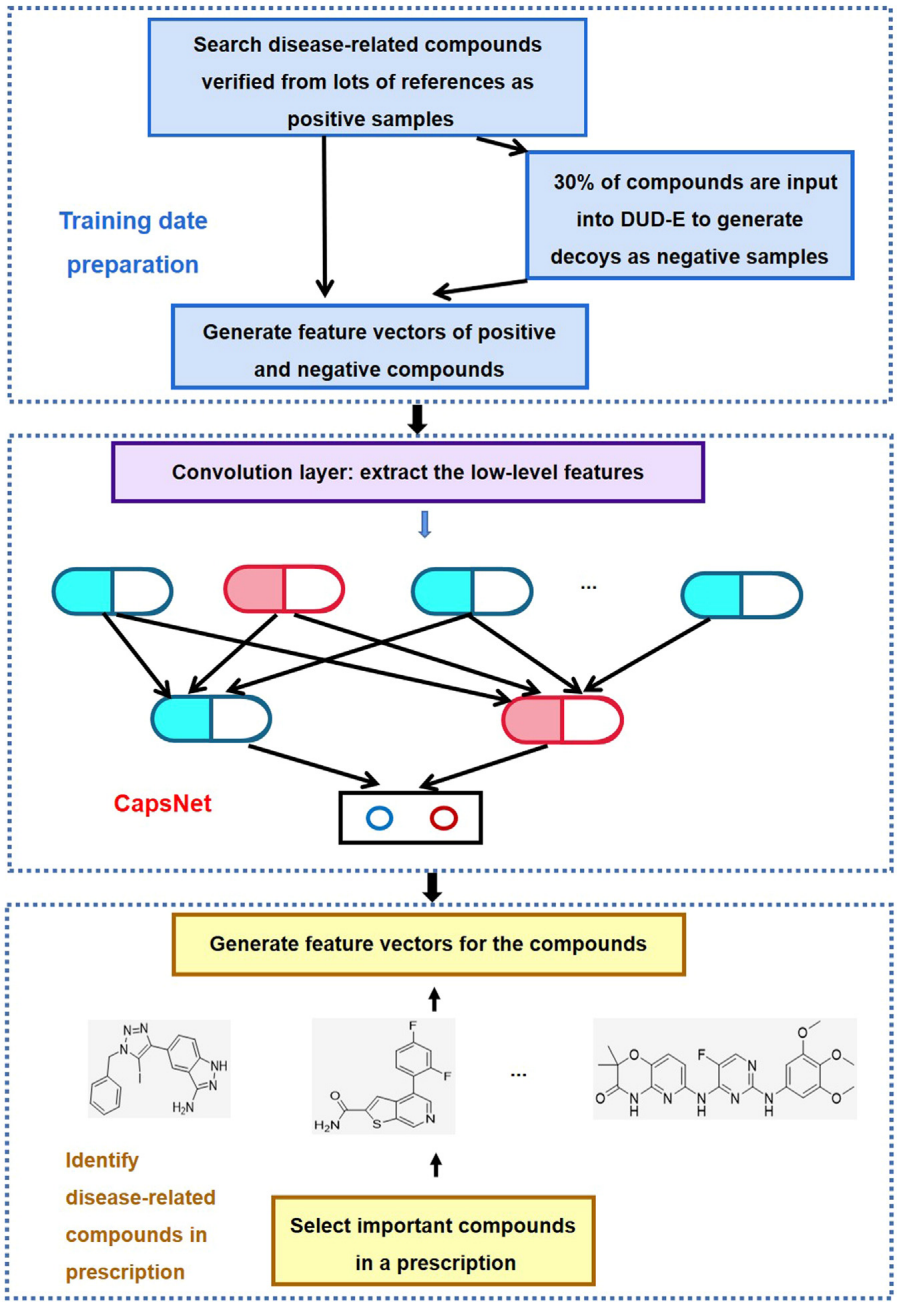
Flowchart of the disease-related compound identification model based on CapsNet Reproduced with permission from ref.^57^ Copyright 2022 Oxford University Press.

Recently, a novel CapsNet-based model for identifying disease-related compounds was proposed.^57^ As far as we know, this is the initial endeavor to utilize CapsNet in a network pharmacology-related domain. The performance of the proposed model was assessed using the identification of pneumonia-related compounds as a case study. The research process is summarized in Figure 7. Comparing pneumonia-related compound identification across five methods (CapsNet, Support Vector Machine (SVM), Random Forest, gcForest, and forgeNet), CapsNet emerged as the most accurate in identifying such compounds, outperforming the other four methods. The above studies confirmed CapsNet’s potential ability to utilize small datasets in the prediction of molecular properties in drug discovery and design.

### Extraction of interactions among entities

#### Chemical-protein interactions

Accurately recognizing the relations between chemicals and proteins is a major task, which plays a vital role in precision medicine, and basic biomedical research, highlighting the significant application of AI in drug discovery.^102^^,^^103^^,^^104^ Scientists have explored and discovered various associations between chemicals and proteins in biomedical research.^105^^,^^106^^,^^107^ The growing volume of biomedical data and advancements in AI enable the efficient detection of these relations. Due to their exceptional capability to autonomously comprehend both semantic and syntactic details, deep learning algorithms have emerged as a promising method for extracting features of chemical-protein interactions from biomedical literature.^105^^,^^108^^,^^109^^,^^110^^,^^111^ Despite the considerable success of many deep learning-based predictive methods, several challenges persist. One major issue is that the performance of these models is highly dependent on the quality of the datasets used. Additionally, certain fundamental operations in deep learning, such as average pooling and max pooling, struggle to preserve precise spatial relationships among complex features and often lack adequate guidance for classification tasks. In order to address these issues, Sun et al. introduced a fresh approach, known as BERT-AttCapsule, for the extraction of chemical-protein interactions.^19^ In this model, the activity vectors of a capsule were used to represent the instantiation parameters for chemical-protein relation types, in which, the orientation of the activity vector represent the properties of the chemical-protein relation types, and the length described its existence probability. The study showed that BERT-AttCapsule can be superior to other state-of-the-art methods in identifying sophisticated and interleaved features. For BERT-AttCapsule, its advantages over other methods and its excellent performance mainly result from three operations. First, BERT can capture extensive dependencies across distances and contextual information from input tokens in a bidirectional manner. This results in the acquisition of superior sequence information of higher quality. Second, attention-guided capsule networks can obtain more detailed information on higher-level spatial relationships, leading to enhanced predictions of interactions between chemicals and proteins. Lastly, the self-attention mechanism guides the model to learn different contribution weights of the capsule networks through dynamic routing, thereby enhancing the aggregation performance.

In recent years, an increasing number of studies have begun to explore the application of CapsNet in the recognition of target protein-ligand interactions. However, most of this research remains largely theoretical. In 2024, our research team presented a novel approach that integrates CapsNet-based drug design with traditional target-based molecular docking, successfully identifying a Janus Kinase 2 (JAK2) inhibitor with a unique scaffold.^112^ This inhibitor demonstrated promising *in vitro* activity for the treatment of myeloproliferative neoplasms (MPN). Our study provides significant empirical support for the continued application of CapsNet in the design of small molecule targeted drugs. For target-based drug design, the structures of different compounds can exhibit significant variability. CapsNet has the advantage of integrating heterogeneous information and extracting potentially effective features, which enhances predictive accuracy. So, the application potential of CapsNet in predicting drug-target interactions is increasingly being recognized by researchers.

#### Drug-drug interactions

Drug-drug interactions (DDIs) happen when individuals consume multiple medications simultaneously or in close succession, leading to potential alterations in the potency or side effects of the drugs.^113^ DDIs may not only reduce the drug’s effect but also lead to huge health risks to the patients.^114^ Therefore, detecting DDIs is crucial for enhancing public health safety, and it has garnered growing interest from the biomedical community.^115^^,^^116^^,^^117^ With the rapid development of computing technologies and increase in the number of biomedical publications, plenty of computational methods for extracting DDIs have been introduced.^118^^,^^119^^,^^120^ As a promising approach, various deep learning neural networks have been utilized for extracting DDIs from biomedical texts.^116^^,^^121^^,^^122^^,^^123^ In 2021, Wang et al. introduced an innovative technique featuring an attention-based CapsNet for DDIs relation classification. This model employed an improved sliding-margin loss function instead of the traditional margin loss approach.^20^ When compared to other classic deep learning models, this novel model demonstrated superior performance. Similar to the above work, the dynamic routing mechanism was employed to transfer information from low-level capsules to high-level capsules in the work of Zhang et al.^124^ To further validate the performance of the proposed method, a comprehensive comparison was conducted against four traditional DDIs extraction methods. The model proposed showed better performance when compared to other methods. The results suggested that utilizing dynamic routing within the capsule network has the potential to greatly enhance the model’s performance.

#### RNA-protein interactions

Research has accumulated findings indicating that RNA-protein interactions play an important role in cellular homeostasis, and perturbation of the interactions of RNA-binding proteins can lead to cellular dysfunction and disease.^125^^,^^126^ Identifying RNA-protein interactions is essential for understanding the relationship between proteins and disease. There are many novel predictive tools for distinguishing RNA-protein interactions relating to the successful application of CapsNet. Song et al. developed a hybrid deep learning architecture called AC-Caps, which integrates an attention mechanism, CNN, and CapsNet, to predict RNA-binding protein binding sites on LncRNA.^58^ They discovered that dynamic routing allows the model to more accurately represent the relationships between local features surrounding the RNA binding site and the overall protein structure. Another example that utilized CapsNet for identifying LncRNA-protein interaction (Capsule-LPI) was introduced by Li et al.^25^ Under identical feature sets and test conditions, Capsule-LPI demonstrated superior performance compared to other deep learning architectures, including fully connected layers, CNN, long short-term memory, and deep stacking network architecture. A different model was presented by Wang et al. for circular RNA (circRNA) and protein interactions. The model, named circRB, was a modified version of CapsNet and aimed to recognize the sequence preferences of circRNAs interacting with RNA-binding proteins.^59^ Consistently, these findings have shown that methods constructed on CapsNet are effective, achieving higher prediction accuracy compared to other methods in similar conditions. Thus, CapsNet has contributed to better understanding and discovery of the structures and functions of drug targets.

## Challenges and future perspectives

CapsNet has shown promising results in drug discovery, such as better generalization to novel data, decreasing computational cost through accelerated convergence and fewer parameters for training, and improved predictive performance for sparse data. While CapsNet has shown effectiveness in various key stages of drug discovery and its related components, the process itself is complex and the current CapsNet model may not be fully tailored to address all facets of drug discovery optimization. So, it is impossible to be sure that CapsNet could be compatible with each task in drug discovery. Besides, there is still no uniform metric or benchmark dataset for assessing CapsNet’s applications in drug discovery. CapsNet is a different approach for addressing small-data issues by enhancing the model’s capacity across different tasks. However, it remains in its early stages, with several limitations yet to be explored. The major drawbacks of the basic CapsNet have been summarized in previous reports.^127^^,^^128^ When it comes to applying CapsNet to drug discovery, numerous obstacles still need to be overcome.

The first challenge is how to further employ the CapsNet algorithm to address more crucial issues in various tasks of drug discovery as cost-effectively as possible. Design of new drugs is seen as a practical way to reduce the vastness of chemical space, to generate novel compounds, and to optimize hit-to-lead.^129^ Most traditional *de novo* drug design methods heavily rely on existing knowledge. Knowledge-based methods yield new structures by replacing or adding molecular fragments, potentially skewing the exploration space and excluding other configurations beyond the knowledge sphere.^130^ In recent years, generative models based on deep neural networks have emerged as a promising option for *de novo* drug design without any prior chemical knowledge.^131^^,^^132^^,^^133^^,^^134^ So far, several bioactive compounds have been discovered by deep learning-based methods.^135^ Until now, CapsNet has not been exploited to develop generative models in the field of drug research. Given its superior performance in extracting valuable features and predictions, CapsNet might be implemented as a feature extractor and classifier for sampled molecules, and it could be integrated with a generative model. Researchers have concentrated on enhancing the predictive capability of CapsNet for specific tasks, aiming for increased accuracy or reduced errors compared to other methods under similar conditions. However, obtaining novel hits and leads in drug research through CapsNet remains challenging. We suggest that the performance of CapsNet in drug discovery could be improved by combining advanced algorithms and additional feature extractors.

The subsequent challenge lies in accurately elucidating the CapsNet technique within the context of drug discovery implementation. CapsNet’s predictive performance can vary depending on the data sources and target tasks, and the underlying causes for this variation remain unclear. The impressive performance of various CapsNet models can be primarily attributed to the use of capsules or vector neurons. Nevertheless, as an emerging architecture, CapsNet remains somewhat enigmatic and requires further exploration within drug discovery application domains. Considerable additional research is necessary to fully comprehend its attributes. In a study by Wang et al., a predictive model based on CapsNet was proposed to identify protein PTM sites, in which biological meanings of the capsules were analyzed to interpret what was learned by the capsules.^31^ Khanal et al. introduced an extended version of the CapsNet architecture, termed DeepCap-Kcr, designed to identify and investigate protein lysine crotonylation sites. The study also explored the rationale behind the model’s predictions and their biological implications.^55^ Through visualization of the characteristics detected by the CapsNet-based model, these investigations analyzed the internal distribution of data pertaining to motif identification. Despite this, many questions regarding CapsNet-based models for drug discovery are difficult to answer. For example, as an important procedure in CapsNet, the possible biological/chemical meanings related to the dynamic routing algorithm have not been defined explicitly in the related literature yet. Moreover, we are still unable to accurately define the composite of substructures and/or characteristics, the key elements for the model, and the difficult examples for categorization. With the advancement of model interpretation techniques, it is anticipated that this issue will soon be addressed. We will thoroughly explore the integration of capsule neurons, which capture the instantiation parameters of entities, with modern model interpretability techniques such as model visualization and attention mechanisms. This integration aims to enhance the interpretability of CapsNet models. One suggestion is to employ visualization and feature activation mapping techniques with the CapsNet model. By visualizing the specific features of each activated capsule, we can intuitively showcase how the model comprehends and interprets the input data. For instance, techniques such as Class Activation Mapping (CAM) or Gradient-weighted Class Activation Mapping (Grad-CAM) can be used to identify which features of the input data contribute most to the predictive results.^136^^,^^137^ By tracking and visualizing the paths of activated capsules during the decision-making process, researchers may directly observe which combinations of features have influenced the model’s decisions. This strategy contributes to understanding how CapsNet performs rigorous logical reasoning from low-level to high-level functions to some extent. The second strategy involves integrating the self-attention mechanism within the CapsNet framework. By incorporating the self-attention mechanism into the CapsNet architecture, the model’s ability to concentrate on the most pertinent sections of the input data can be improved. By weighing the relationships and significance among various capsules, self-attention can enhance the model’s accuracy and interpretability when confronted with complex or variable inputs.^138^^,^^139^^,^^140^^,^^141^ Another proposal is to develop a cross-capsule attention mechanism specifically designed for the CapsNet framework, which would enable dynamic regulation of the information flow amongst various capsules. This measure could assist the model in better distinguishing between key features and noise within the input data, thereby offering a clearer perspective for interpreting the model. The third approach is to integrate an interpretable embedding layer into the CapsNet architecture. Before the CapsNet input layer, an interpretable embedding layer is introduced. Its purpose is to transform the raw input into a format that the model can process and understand more effectively.^142^ This layer can utilize interpretable feature learning methods, such as sparse coding or dictionary learning, to enhance the transparency and interpretability of the entire model. We hypothesize that the outlined strategy might not only maintain the superior performance of CapsNet but also amplify its interpretability. This enhancement has the potential to significantly expand its utility in the field of drug discovery. It is important to note that the effectiveness and implementation of these methods can vary significantly depending on the application and the specific architecture being used. Thus, rigorous validation and fine-tuning is essential during both the design and implementation.

The obstacles previously discussed can be conquered soon with substantial investments in time and capital along with simultaneous technological progress. Rationalization of drug design technology driven by deep learning will achieve substantial progress soon. CapsNet has great potential in revolutionizing virtual screening through deep learning. Traditional high-throughput computational approaches using deep learning often face a common challenge: a significant issue with false positives, resulting from an imbalance in the positive and negative data within the training dataset. To improve the interpretation of information obtained from limited experimental data and to enhance task-specific performance, CapsNet effectively identifies and emphasizes critical information from receptors, ligands, and interactions, particularly for minority classes in imbalanced datasets. Additionally, it should be admitted that the problem of a low data problem, basic to drug research, has not been completely solved. Table 4 summarizes the pros and cons of the typical methods in small-size dataset. From the table, it is evident that each method has its unique features, making it difficult to determine which one could effectively address all the challenges associated with small dataset problems in drug discovery tasks. We endeavored to create a pioneering model that combines CapsNet with multitask learning to predict toxicants. Our findings indicate that multitask CapsNet was able to extract more valuable information for the minority class in the training set compared to single-task CapsNet. It is reasonable to assume that the low data of negatives in high-throughput screening process will be further improved by a combination of CapsNet, other methods that work well on small datasets and the data augmentation techniques.

**Table 4:** Comparison of various methods that perform effectively on small datasets in drug discovery

Method	Pros	Cons
CapsNet	It can overcome information loss caused by the pooling strategy of convolutional neural network. It has powerful ability in extracting features.	It is unable to identify two very close entities, namely “crowding problem”. Its inner loop procedure is slow.
Transfer learning (a type of learning paradigm, including multitask learning, meta-learning, continual learning etc.)	It can learn generalizable knowledge from other related tasks.	It lacks interpretability. Negative transfer needs further systematic analyses.
One/few-shot learning	It has rapid convergence and good generalization ability.	Unreliable empirical risk minimizers are the core issue. The catastrophic forgetting is a major limitation in essence.
Zero-shot learning	It can recognize object classes not seen at the training stage.	It requires a substantial quantity of precise descriptors. The noisy or biased attribute annotations can lead to poor performance.

Despite the advantages of CapsNet models in handling small datasets, achieving high performance requires careful and high-quality data preprocessing. Subsequently, we will discuss the application of CapsNet in drug discovery, emphasizing the methods for data preparation and preprocessing. First and foremost, the data must undergo cleaning and normalization. Ensuring dataset integrity and removing irrelevant attributes are vital for developing high-quality models. For example, normalizing bioactive and inactive data helps CapsNet models learn and converge more quickly. When dealing with image data, standardizing pixel values is crucial. Similarly, for numerical data, scaling all features to a consistent range is essential for achieving optimal model performance. Second, data augmentation techniques can be effectively utilized to enhance suitable data. For image datasets, methods like rotation, scaling, and minor modifications can significantly boost CapsNet’s ability to recognize patterns from various angles and scales. To handle the complex hierarchical structures of pharmaceutical molecules, synthetic data can be generated to enrich the dataset, providing an alternative approach.^143^ Third, the data should be characterized structurally based on the actual situation. CapsNet’s ability to represent hierarchical relationships allows it to emphasize these structures in the data effectively. For instance, with small molecular compounds, CapsNet can hierarchically represent molecules by focusing on the relationships between chemical bonds and functional groups. Thus, converting raw data into a structured format can effectively emphasize the key features and relationships pertinent to drug discovery tasks. Fourth, it is necessary to serialize and refine the sequential data. It is crucial to properly serialize sequential datasets, like protein sequences, and ensure they maintain the same scale when input into the CapsNet model. This is vital for preserving integrity across various parts of the sequence. Specialized techniques are employed for data preprocessing. For complex molecules, utilizing a graph-based representation to learn the relationships between atoms and molecular structures has the potential to fully leverage CapsNet’s advantages in image recognition. Dimensionality reduction methods like Principal Component Analysis (PCA) are applied to condense data without sacrificing vital information, thereby enabling CapsNet to process precise information more swiftly and efficiently.^144^^,^^145^^,^^146^ Fifth, diversity of data must be ensured. To strength the generality of the model, the limited training dataset should include various forms of data that might be encountered in real-world. Collectively, careful preparation and processing of raw data, along with the selection of suitable hyperparameters that capture complex patterns and hierarchical relationships specific to each task, will enhance the performance of CapsNet in drug discovery.

To investigate the potential applications of CapsNet in drug discovery, we conducted a systematic comparative analysis with conventional deep learning algorithms (see Table 5). CNN is proficient in image processing, effectively extracting spatial features through local receptive fields and weight sharing. However, their fixed feature representation renders them susceptible to image deformations, rotations, and translations, which can impair classification accuracy. In contrast, CapsNet employs a capsule structure to capture spatial relationships between features more effectively, enhancing robustness to object deformations and pose variations. Its dynamic routing mechanism facilitates the transfer of information from lower-level to higher-level capsules, improving the model’s capacity to comprehend complex patterns. While Artificial Neural Network (ANN) provides some flexibility, their relatively simple structure limits their ability to manage complex data and typically requires large training datasets to mitigate overfitting. CapsNet, however, excels with smaller datasets due to its efficient feature representation. Despite its higher computational complexity, CapsNet’s potential for few-shot learning offers advantages in specific applications, including high-throughput drug screening. GNN focuses on processing graph-structured data, demonstrating strong performance particularly in social networks and recommendation systems. GNN is capable of capturing relationships between nodes and information about graph structure, but they may encounter challenges when handling high-dimensional features.

**Table 5:** Comparison of the advantages and disadvantages of CapsNet and three representative deep learning methods

Method	Pros	Cons
CapsNet	It utilizes capsule units to capture the spatial relationships between features, improving capabilities for modeling these relationships. It exhibits greater stability when dealing with input disturbances, enabling it to effectively differentiate between various objects. It effectively reduces the number of parameters in the model, thereby lowering the risk of overfitting.	The dynamic routing algorithm of CapsNet has a high computational cost, resulting in slower training and inference speeds. It is still evolving in both theoretical and practical applications, and it lacks widespread practical experience.
CNN	It effectively extracts local features, making it particularly suitable for processing grid data such as images. By sharing parameters, the number of parameters is reduced, enhancing the efficiency of model training. It is widely used in fields such as image recognition and object detection, with developed frameworks and numerous examples.	It lacks sensitivity to object deformation and changes in pose. Its ability to capture spatial relationships between features is limited. Its performance relies heavily on a large amount of labeled data.
ANN	It can be applied to solve a variety of problems, including classification, regression, and structured data tasks. Its structure is relatively simple, making it easy to implement and debug. It has a relatively fast training speed.	It has limited ability to learn from complex data, such as images and audio. It struggles to effectively capture features of high-dimensional data. It is prone to overfitting when dealing with small sample sizes.
GNN	It is particularly well-suited for handling various types of graph-structured data. It can combine local features of nodes with global graph structure information effectively.	It has high computational complexity and requires longer training time. It features a complex model design and requires tuning a significant number of hyperparameters. Its theoretical foundations and practical experience are still evolving.

In summary, CapsNet’s unique strengths in complex data handling and feature relationship capture, alongside its integration with other deep learning methods, could pave the way for new research directions in drug discovery.

The next promising subdomain is in lead optimization, which is one of the most complicated procedures in the early stage of drug discovery and highly dependent on the experience and skills of medicinal chemists. Once the compounds that can block or activate the target protein have been confirmed by a preliminary biological evaluation, structures are further modified to improve the activity and specificity against the target along with optimized pharmacodynamic, pharmacokinetic, and toxicological properties. As capsules can capture new viewpoints through linear transformation parameters, CapsNet exhibits superior generalization capabilities compared to commonly used deep learning techniques. Thus, CapsNet excels in accurately classifying previously unseen data. We can apply CapsNet to various tasks of the lead optimization step. Currently, generative models are an important approach to design novel compounds with optimal values for solubility, pharmacokinetic properties, bioactivity, and other parameters, and capsule layers could be integrated with generative adversarial models to serve as outstanding discriminators. After that, the most efficient and cost-effective synthetic route required for producing the optimal new molecules could be identified. CapsNet’s ability to model hierarchical relationships within internal knowledge representations may enable it to extract more useful information, even when reaction rules lack logical relationships. Recent studies suggest that CapsNet shows significant potential in various areas of drug discovery. With further modifications, it could feasibly address complex challenges and unresolved issues related to drug discovery and design soon.

## Acknowledgments

This research received funding from the 10.13039/501100001809Natural Science Foundation of China under grant number 82374073, alongside support from the Sichuan Science and Technology Program of China with project codes 2023NSFSC1692 and 2022YFS0614, as well as the Luzhou Science and Technology Program of China, ref. 2022-SYF-46, and support by the Yibin Science and Technology Planning Project of China with codes 2022NY020, 2021XZXNYD01, 2021ZYY009 and 2021ZYY005.

## Author contributions

Y.W. conducted a comprehensive review of the relevant literature and drafted the manuscript, while B.W. provided editorial assistance and created various figures. J.Z., A.W., Y.L., and Y.W. contributed to the review and revision of the manuscript. J.L. and J.W. supervised and facilitated the entire process. All authors have reviewed and approved the final version of the manuscript.

## Declaration of interests

The authors declare no competing interests.
